# A time for optimism in the dementia field

**DOI:** 10.3389/frdem.2022.1038003

**Published:** 2022-10-18

**Authors:** William L. Klein

**Affiliations:** Departments of Neurobiology and Neurology, Northwestern University, Evanston, IL, United States

**Keywords:** dementia, Alzheimer's disease, neurodegeneration, drug discovery, progress

Now is a great time for optimism in the dementia field. Clinical trials are underway for next generation therapeutics focused on familiar targets; new targets are being revealed by discoveries of novel mechanisms; re-kindled enthusiasm for the field is emerging in pharma; and NIH and other funding agencies are increasing their support for dementia research. All this despite the recent past's chronic story of clinical trial disappointments and the withdrawal of Aduhelm, the only new treatment for Alzheimer's approved in the last 20 years. It has become obvious to society that dementia not only has a heartbreaking impact on individuals and their families but is a titanic problem for society itself. Without effective treatments, by 2050 the cost of Alzheimer's in the US alone is predicted to reach a trillion dollars a year^1^.

When we look back on challenges already met, we see a strong foundation for future progress. At its base is our altered perception of dementia. Two thousand years ago Virgil wrote that “*Aging steals from us all things, even the mind*,” but most of us no longer accept this view. Dementia in its various forms is a malady, not a natural part of aging, demonstrated over a century ago by Alois Alzheimer in his study of Augusta Dieter. Social awareness of this principle, thanks to efforts of charities and foundations, has galvanized the public to demand progress. And progress has come. Searches for molecules that constitute the pathologies have been remarkably successful. We understand how good molecules turn bad, how they can be linked to neurological deficits, and what cell and molecular mechanisms underlie the damage caused to brain cells. Beyond specific toxins, we appreciate the breadth of pathological changes that accrue in dementia, and we have a new understanding of the stereotyped progression of pathology from one brain region to another. Passing these milestones has revealed multiple plausible targets for therapeutics, and the first steps have been taken, not only for treating disease consequence but for testing approaches that could lead to disease modification.

## Challenges ahead

A detailed and practical perspective on today's challenges can be found in the NIA statement of priorities for research into Alzheimer's and Alzheimer's-Related Dementias [www.alzheimers.gov > research-activities]. Broadly, challenges at our frontier are of two sorts. Some lead us to “*go boldly where no one has gone before*^2^”. New phenomena will be discovered, and new mechanisms and targets will be revealed. Findings at this frontier, increasing almost exponentially, lead to fresh questions and opportunities, providing excitement about the future. Some challenges are perhaps more taxing. They require us to deal with phenomena that have been investigated for decades – the roles, e.g., of amyloid beta and tau in Alzheimer's and related dementias.

### Toxins in the brain

Some argue that less effort be devoted to studies of Aβ toxicity, and to some extent tau toxicity, too. The field, after all, is littered with clinical failures. But pathological forms of Aβ and tau, capable of triggering brain damage, invariably manifest in the brains of persons demented by Alzheimer's and related diseases. Decades of research show the oil is there – it's the drilling that has gone awry. A big plus in targeting toxins for disease-modifying therapeutics is that targeting pathogenic rather than physiological molecules should be less prone to side-effects. And it's advantageous to build on successes, even if minor. Aduhelm is an example: Aduhelm is expensive and gives just minor benefits, but it serves as a clue, and we should use it to drill down. The challenge is to find new ways to approach the familiar. There are many questions and possibilities. What molecular modifications turn normal proteins into toxins? Will new generations of antibodies be found? Can we find a better means of antibody delivery, maybe gene-based? Can regulatory dysfunctions responsible for toxin buildup be identified - and corrected? Can holistic ways be found to keep toxin levels low? Can vaccines, shunned for two decades, be resurrected safely and effectively, using new adjuvants and antigens? To move the needle, persistent creativity is needed. We might not totally agree with Einstein when he said, “*It's not that I'm so smart, it's just that I stay with problems longer,”* but his point on persistence is well-taken.

### Mechanisms that destroy

While targeting toxins rather than physiological molecules would be ideal, success could continue to prove evasive. A parallel challenge thus is to find targets underlying other facets of dementia-causing pathology. Current research is pursuing pathways leading to inflammation, oxidative and ER stress, dysfunctional autophagy, and insufficient energy metabolism. These are rich veins of current research, but the challenge is to find targets that can be safe as well as disease-modifying. Age being the greatest risk factor for dementia, a key challenge remains to elucidate the relationship between normal aging mechanisms and dementia-causing mechanisms. Compared to normal aging, is age-onset dementia an “aggressive” form of aging (a quantitative issue) or is it “different” (a qualitative issue)? And what can we learn from the similarities and differences between major dementias (AD, vascular, frontal-temporal, and Lewy body)? There likely are commonalities. What is the significance of PART (primary age-related tauopathy) – what is its origin, and how does it contribute to dementia? Etiological mechanisms overall are poorly understood – we can wonder why AD is more common than all the dementias put together. Relationships between risk factors and pathogenesis still are uncertain. And what causes the pathological molecules of dementia to manifest selectively in the brain? We have learned that a stereotypic progression of pathology occurs in AD; the next challenge is to understand the mechanism underlying this progression. And why does AD start “somewhere?” What if that part of the brain was prevented from “starting” – would another part of the brain take over and start the disease in a new location?

### Mechanisms that protect

Is dementia inevitable? With sufficient age, will everyone become demented? Some studies say yes, but there is emerging optimism in the challenge to understand and learn from the success of super-agers. How can we explain their cognitive success? It has been suggested that the cornerstones of mechanisms that protect against dementia comprise *Resistance, Resilience, Reserve, and Compensation* (Montine et al., 2019). Some resistance mechanisms are evident, for example the role of ApoE2 as an anti-risk factor, or the Icelandic mutation in APP, a mutation that retards the onset of Alzheimer's. There is much more to be learned, however, and studies of super-agers are likely to help us elucidate mechanisms that protect. A new direction may come from discoveries that molecules linked to Alzheimer's dementia (Aβ oligomers and hyperphosphorylated tau) sometimes are transiently expressed to serve physiological functions, helping create the normal circuitry of the brain during neural development and helping resist viral infections in the adult brain (Eimer et al., 2018; Bartley et al., 2022). The challenge is to discover the regulatory mechanisms that turn on and off expression of these dementia-linked molecules.

### Better preparation for clinical trials

Roughly 20 billion dollars have been spent on clinical trials that were unsuccessful. Preclinical evidence always anticipated success. The challenge is to develop better models with realistic predictive value. Transgenic models opened the door to investment in drug discovery but non-transgenic models that better match the dementia phenotype are needed. Useful companion diagnostics are also needed – with readouts that quantify disease-modifying efficacies of investigational new drugs at the molecular level. Ultrasensitive assays for dementia-causing toxins in blood would be ideal, and the possibility of imaging retina pathology as a window to disease-onset and progression has appeal. As more is established regarding pathogenic protein structures, we can envision new diagnostic assays with high signal-to-noise readouts to better guide drug development, ultimately leading to precision medicine for treating dementia in its various forms.

### Pragmatism

Thirty years ago, Khachaturian and Coleman pointed out that “*slowing the progression of the disease by only one year would be extremely cost-effective*” (Coleman, 1992; Khachaturian, 1992). One may hope that choosing the right behavior could help slow the onset and progression of dementia. The literature suggests that exercise is beneficial, and, with more research, it is likely that various dietary factors also will be found beneficial. What's good for your heart seems to be good for your brain. The challenge is to encourage individuals to act on what is good for them – never easy. Socialization also may help, although whether the link is causative, or correlative, needs more clarity. A related challenge is to give patients better treatment – to elevate awareness of a dementia patient's humanity. Better caregiving can improve quality of life and lead to cost savings – potentially a positive loop. While each of these factors may have only a small effect, they can make a difference now because they can be acted on now.

### Integrated knowledge base

A PubMed search of “dementia” yields a quarter-million hits. It would take half a year just to read the titles. Linus Pauling said, “*You can't think without facts*,” but more to our challenge is a point made by Henri Poincare, “*Science is built up of facts, as a house is with stones. But a collection of facts is no more a science that a heap of stones is a house*.” It would be of value to integrate the cogent facts about dementia into a comprehensive knowledge base, organized and regularly updated to provide a foundation for creative thinking. This is perhaps our greatest intellectual challenge.

## Final points

The major forms of dementia comprise Alzheimer's, vascular, frontal-temporal lobe, and Lewy body. A half dozen less prevalent dementias also occur. Alzheimer' disease has garnered the most attention because its prevalence is greater than the other dementias combined. Research growth, however, is taking place in all areas. This Frontiers journal will provide a centralized resource that covers all types of dementia. Our expectation is that fresh insights into one type of dementia will inform new lines of study into the others.

As investigational new drugs fail, and fail consistently, it is easy to forget the amazing progress that has accrued. We're impatient, which is good, but we should appreciate all that has come before, and use it for inspiration and motivation. This is a time for resilience and optimism and a time to remember how success will ultimately be reached. Great science occurs at the frontier, and we are privileged to be pioneers. As Theodore Roosevelt said, “*Far and away the best prize that life offers is the chance to work hard at work worth doing*.”

## Author contributions

The author confirms being the sole contributor of this work and has approved it for publication.

## Conflict of interest

Author WK is a founder and scientific advisory board member of Acumen Pharmaceuticals.

## Publisher's note

All claims expressed in this article are solely those of the authors and do not necessarily represent those of their affiliated organizations, or those of the publisher, the editors and the reviewers. Any product that may be evaluated in this article, or claim that may be made by its manufacturer, is not guaranteed or endorsed by the publisher.

## Footnotes

^1^(www.alz.org > facts-figures).

^2^(Jean-Luc Picard).
